# Novel Nanocomposite of Carbonized Chitosan-Zinc Oxide-Magnetite for Adsorption of Toxic Elements from Aqueous Solutions

**DOI:** 10.1021/acsomega.4c06541

**Published:** 2024-11-20

**Authors:** Dalia A. Ali, Ganna G. Ismail, Ahmed I. Osman, Salwa B. Alreshaidan, Ahmed S. Al-Fatesh

**Affiliations:** † Department of Chemical Engineering, The British University in Egypt, El-Sherouk City 11837, Egypt; ‡ School of Chemistry and Chemical Engineering, Queen’s University Belfast, David Keir Building, Stranmillis Road, Belfast, Northern Ireland BT9 5AG, U.K.; § Department of Chemistry, Faculty of Science, King Saud University (KSU), P.O. Box 800, Riyadh 11451, Saudi Arabia; ∥ Department of Chemical Engineering, College of Engineering, King Saud University (KSU), P.O. Box 800, Riyadh 11421, Saudi Arabia

## Abstract

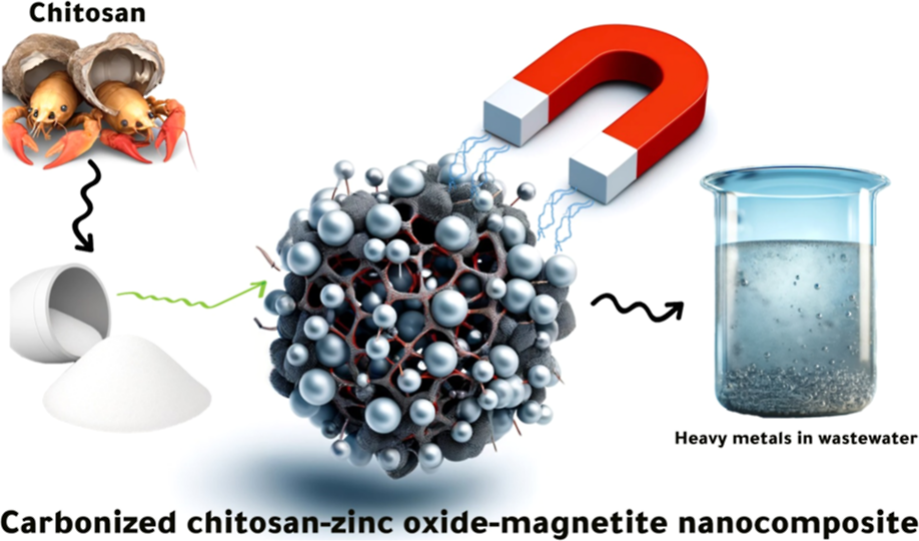

Herein, a novel nanocomposite (carbonized chitosan-zinc oxide-magnetite, CCZF) was developed to effectively remove toxic elements in water remediation. Combining the high adsorption capacities of chitosan with the magnetic properties of magnetite and the chemical stability of zinc oxide, the combination of these unique properties makes it an efficient and versatile material that offers a sustainable solution for water purification. The (CCZF) nanocomposite was synthesized through the coprecipitation method and characterized using various techniques, including scanning electron microscopy (SEM), transmission electron microscopy (TEM), Brunauer–Emmett–Teller (BET) analysis, X-ray diffraction (XRD), Fourier transform infrared (FTIR) spectroscopy, and zeta potential analysis. The results showed impressive maximum adsorption capacities of 891.34 mg/g for Ni^2+^, 1269.35 mg/g for Co^2+^, and 1502.67 mg/g for Cu^2+^, fitting well with a modified Langmuir isotherm model. The adsorption process was spontaneous and endothermic, characterized by low positive enthalpy (Δ*H*) values ranging from 10.95 to 34.9 kJ/mol, indicative of the physical adsorption mechanism. Additionally, the nanocomposite demonstrated good reusability over multiple adsorption and desorption cycles. This research highlights the potential of the (CCZF) nanocomposite as a highly efficient, reusable adsorbent for the removal of toxic elements from aqueous solutions, contributing significantly to environmental remediation efforts and pollution control.

## 1 Introduction

Water is essential for life, serving many purposes, including drinking, agricultural irrigation, industrial production, energy generation, household needs, and animal feeding.^1,2^ It also plays a critical role in maintaining the stability of our environment and ecosystems.^3^ Despite its importance, fresh water is incredibly scarce, making up only 3% of the Earth’s total water, with the remaining 97% being salt water found in oceans and seas.^4^ The world is facing a water crisis worsened by the increasing demand for fresh water and the contamination of water sources. Industrial wastewater, in particular, is a significant contributor to water pollution, containing hazardous contaminants like toxic elements.^5^ These toxic elements, such as copper (Cu), zinc (Zn), lead (Pb), chromium (Cr), iron (Fe), mercury (Hg), cadmium (Cd), cobalt (Co), arsenic (As), and nickel (Ni), are among these contaminants.^6,7^ Cu is essential for various metabolic functions; however, elevated levels can lead to toxicity.^8,9^ Excessive accumulation of Cu in the body is associated with several health issues, including various forms of cancer, gastrointestinal disorders, skeletal alterations, and potentially fatal poisoning.^8,9^ Co, recognized as a vital nutrient, is a key component of vitamin B12 and is crucial for optimal thyroid function.^8,9^ It also plays a significant role in regulating blood pressure and offers various health benefits.^8,9^ Nevertheless, excessive exposure to Co can have detrimental effects on human health, particularly affecting the respiratory and cardiovascular systems, as well as causing skin irritations.^8,9^ Ni, although required in trace amounts for the synthesis of red blood cells and the activation of certain enzymatic processes, can become toxic at higher concentrations.^8,9^ Prolonged exposure to elevated Ni levels may lead to cellular damage, liver and heart complications, and a reduction in body weight. Ni toxicity is also linked to impaired cell growth, an increased risk of lung cancer, and damage to the nervous system.^8,9^ Sources of toxic element contamination include natural, residential, agricultural, and industrial activities, as well as miscellaneous sources like incineration, medical waste, landfills, and traffic emissions.^4^ To mitigate these risks, various treatment methods have been developed to remove toxic elements from industrial wastewater. These methods include membrane, chemical precipitation, and adsorption methods.^5,10^ Membrane processes, which include ultrafiltration, microfiltration, distillation, reverse osmosis, nanofiltration, forward osmosis, electrodialysis, and liquid membrane technologies, are widely used for treating wastewater.^11^ These processes are influenced by factors such as pore size, hydrophilicity, pore distribution, surface charge, solution flow, and the presence of functional groups.^12,13^ The interpretation of the water production rate and heavy metal removal efficiency of the entire membrane process significantly depends on these variables. Its selectivity and flux rate typically govern the membrane’s performance. One of the most effective and mature methods for determining precipitation status is chemical precipitation (coagulation precipitation). As a result, dissolved toxic elements are transformed to solid particles that can be sedimented more easily. Through reagent coagulation (coagulant), toxic elements are precipitated by changing the pH, electro-oxidizing potential, or coprecipitation.^14,15^ The adsorption mechanism is defined by the physicochemical properties of the adsorbent and toxic elements as well as the operating conditions (temperature, adsorbent amount, pH, adsorption period, and initial concentration of toxic elements). In general, toxic elements can be adsorbed onto the surface of an adsorbent. This approach was found to have low operating costs, a high removal capacity, ease of implementation, and simple treatment procedures based on regenerating the adsorbed toxic elements.^10,15^ Fe particles (often magnetic nanoparticles (NPs) such as Fe_3_O_4_) are present in a particular kind of material matrix called magnetic adsorbents.^16^ Materials such as carbon, CS, polymers, starch, and biomass could be employed as foundation materials. Many magnetic adsorbents, such as zerovalent iron NPs (ZVI NPs), iron oxide hematite (-Fe_2_O_3_), maghemite (-Fe_2_O_3_), magnetite (Fe_3_O_4_), and spinel ferrites, have been used.^16^ The presence of Fe particles in adsorbents is particularly effective at removing toxic elements from effluent.^15^ Previous studies have shown that some adsorbents can remove toxic elements from aqueous solutions with low adsorption capacities. For example, carbon nanotube (CNT)-coated polyamidoamine (PAMAM) was used to remove Co^2+^ with a maximum capacity of 494 mg/g, coconut husk was used to remove Ni^2+^ with a maximum capacity of 404.5 mg/g, and activated carbon was used to remove Cu^2+^ with a maximum capacity of 125 mg/g.^15,17^ Therefore, adsorbents must be developed and improved to be capable of removing high concentrations of toxic elements from aqueous solutions. Existing methods for heavy metal removal often have limited adsorption capacities, which can result in the incomplete removal of contaminants from water sources. This can lead to potential health risks for humans and to environmental damage. Complicated regeneration processes in current methods can also pose challenges, where the materials used for adsorption need to be separated from the toxic elements before they can be reused. This can increase the cost and time involved in the treatment process. Furthermore, some existing methods may have limitations on the types of metals that they can effectively remove. This can be problematic if a wide range of toxic elements needs to be treated in contaminated water sources. Therefore, this study presents the development and characterization of a novel carbonized chitosan-zinc oxide-magnetite (CCZF) nanocomposite, particularly designed for the efficient removal of heavy metals from a ternary-component aqueous system. This research introduces a unique synthesis method that integrates the high porosity of CC, the magnetic properties of Fe_3_O_4_, and the chemical stability of ZnO into a single composite material. The innovative aspect of this work lies in the dual functionality of the (CCZF) nanocomposite, which not only exhibits exceptional adsorption capacities for Ni^2+^, Co^2+^, and Cu^2+^ ions but also allows for easy magnetic separation post adsorption. This dual functionality addresses the common challenge of adsorbent recovery in water treatment processes, enhancing both the efficiency and the sustainability of heavy metal removal from wastewater. The study also provides a comprehensive optimization of the adsorption conditions, making significant strides in environmental remediation technologies by offering a cost-effective and reusable solution for heavy metal ion decontamination.

## 2 Materials and Methods

### 2.1 Chemicals

A variety of analytical grade reagents were used in this study, including ferric chloride hexahydrate (FeCl_3_·6H_2_O, ACS reagent, ≥97%), ferrous sulfate heptahydrate (FeSO_4_·7H_2_O, ACS reagent, ≥99%), chitosan [(C_6_H_11_NO_4_)_n_, high molecular weight, degree of quaternization 30–70%], copper sulfate pentahydrate (CuSO_4_·5H_2_O, ACS reagent, ≥98%), cobalt chloride (COCL_2_, anhydrous, ≥98%), sodium hydroxide (NaOH, ACS reagent, ≥97%, pellets), nickel chloride hexahydrate (NiCl_2_·6H_2_O, 99.999% trace metals basis), zinc acetate [(CH_3_COO)_2_Zn·2H_2_O, ACS reagent, ≥98%], and hydrochloric acid (HCl, 36% v/v). Sigma-Aldrich Company, Germany, provided all of these reagents, with the exception of (C_6_H_11_NO_4_)_n_ (high molecular weight, degree of quaternization 30–70%), NaOH (ACS reagent, ≥97%, pellets), and HCl (36% v/v) obtained from Nano-Gate Company, Egypt. All the solutions were prepared utilizing distilled water.

### 2.2 Equipment

NP imaging was performed using a scanning electron microscope (SEM) (Quattro s, Thermo Scientific, The Netherlands) with high-vacuum imaging of 0.8 nm at 30 kV (STEM) at 30 kV (SE) in high vacuum, 1.3 nm at 30 kV (SE) in low vacuum and ESEM mode, and 3.0 nm at 1 kV (SE). Visualization of the molecules in the prepared nanocomposite at the nanoscale was performed by obtaining 2D images via transmission electron microscopy (TEM) (JEM-1400Flash, JEOL Solutions for Innovation Company, USA). Its optical system can image at an ultrahigh resolution of 0.14 nm while having a magnification range (minimum magnification: 10 times) that can image the entire sample mesh (Φ 2 mm) with a bottom mount camera. The specific surface area, pore size, and particle size were determined by the Brunauer–Emmett–Teller (BET) method (NOVA touch, Quantachrome Company, U.S.A.) with dimensions of 61.6 (width) × 49.2 (depth) × 82.9 (height) cm, electrical potential difference 100–240 V, frequency 50/60 Hz, surface area ranges from 0.01 m^2^/g to no known upper limit, and pore size 0.35 to 500 nm (3.5 to 5000 Å). The chemical composition and crystal structure of the prepared nanocomposites were determined using X-ray diffraction (XRD) (Empyrean – Malvern Analytical Company, The Netherlands) with dimensions of 1400 (width) × 1162 (depth) × 1947 (height) mm and a maximum useable range of −111 < 2θ < 168°. The XRD measuring process involves irradiating a sample of the material with incident X-rays. The next step was to measure the intensities and scattering angles of the X-rays that were scattered by the material. The groups on the prepared nanocomposite were identified using Fourier transform infrared (FTIR) spectroscopy (Vertex 70 RAM II, Germany) in a frequency range of 400 to 4000 cm^–1^ (wavelength from 25,000 nm to 2500 nm), spectral resolution better than 0.4 cm^–1^ (apodized), and a rapid scan >70 spectra/sec at 16 cm^–1^ spectral resolution. The point of zero charge (pzc) of the newly prepared nanocomposite was detected using a dynamic light scattering instrument (ZetaSizer Nano Series (HT), Nano ZS, Malvern Instruments, UK). The ZP device has dimensions of 600 × 320 × 260 (19 kg), with a temperature control ranges from 0 to 90 °C ± 0.1 °C, a standard laser 10 mW, 633 nm, measurement angles (water as dispersant) of 13° + 173°, ZP range > ±500 mV, maximum sample concentration of 40% w/v, minimum sample volume (using diffusion barrier) 20 μL, and maximum sample conductivity of 200 mS/cm. The heavy metal ion concentrations after adsorption were determined using a UV/vis spectrophotometer (UV-5100, Shanghai Metash Instruments Company), with dimensions of 420 × 280 × 180 mm and a wavelength range from 200 to 1000 nm.

### 2.3 Error Analysis

Comparisons of different multicomponent isotherm models were executed via nonlinear regression using the least-squares method. The Marquardt’s percent standard deviation (MPSD), as calculated by eq 1, served as the determinant for the best-fit isotherm equations^18^
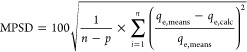
1where *q*_e,calc_ (mg/g) is the calculated adsorption capacity at equilibrium, *q*_e,mean_ (mg/g) is the average of *q*_e,exp_, (*n*) is the number of experimental data points, and (*p*) is the number of factors in each isotherm model.

### 2.4 Preparation of the (CCZF) Nanocomposite

**Figure 1:**
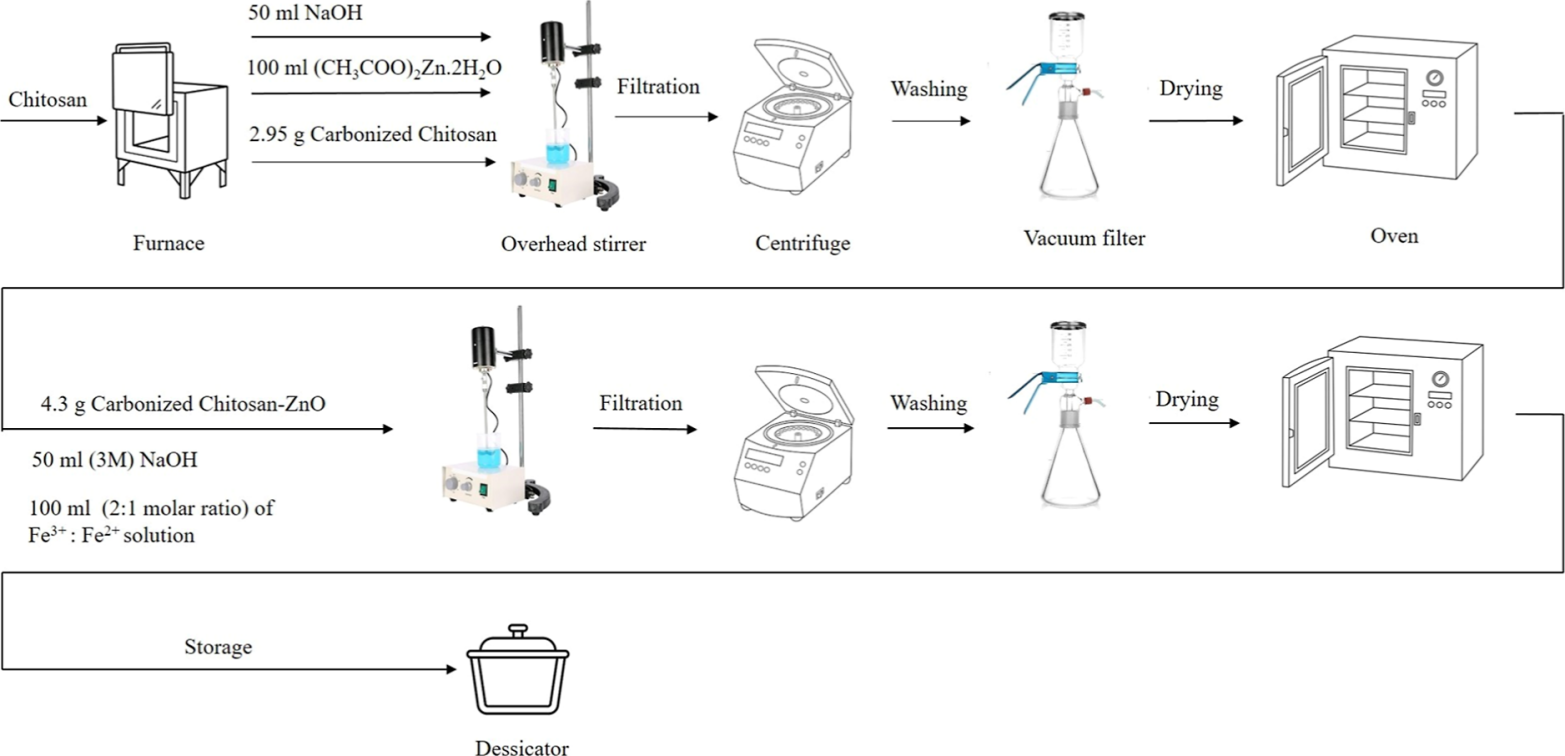
(Carbonized chitosan (CC)-zinc oxide (ZnO)-Fe_3_O_4_) nanocomposite preparation scheme.

CC was used as the precursor for the composite due to its large specific surface area. Carbonization of chitosan took place in a furnace for 2 h at a temperature of 300 °C and a heating rate of 5 °C/min, followed by cooling to room temperature. For the coprecipitation of ZnO on (CC), 2.95 g of the produced (CC) powder was mixed with 100 mL of 1 M zinc acetate, and 50 mL of 2 M NaOH was added to this mixture dropwise, accompanied by mechanical stirring at 500 rpm and heating at 80 °C for 2 h. Filtration was performed via centrifugation at 3000 rpm for 10 min to separate the solid composite CC-ZnO (CC-Z) from the solution, followed by washing with deionized water using a vacuum filter until it reached pH = 7.5 and drying in a dryer at 40 °C. For deposition of Fe_3_O_4_ on the produced composite (CC-Z), 4.3 g of the produced composite (CC-Z) was mixed with 100 mL of a 2:1 molar ratio of Fe^3+^:Fe^2+^, followed by dropwise addition of 50 mL of 3 M NaOH with mechanical stirring at 300 rpm and heating at 85 °C for 2.5 h. The prepared nanocomposite (CCZF) was separated from the solution through filtration using a centrifuge at 3000 rpm for 10 min. Furthermore, the samples were washed with deionized water until they reached neutral pH, dried at 40 °C in a dryer, and stored in a desiccator. Figure 1 shows the preparation scheme of the magnetic nanocomposite (CCZF). Equations 2 and 3 illustrate reactions that occurred during the synthesis of the novel magnetic nanocomposite (CCZF):

- *Zinc acetate reaction with NaOH to produce ZnO*

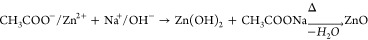
2

- *The production reaction of* Fe_3_O_4_

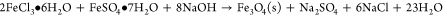
3

### 2.5 Batch Experiments

Adsorption of heavy metal mixtures Cu^2+^, Co^2+^, and Ni^2+^ from aqueous solutions was carried out in batches at pH = 9, which was adjusted with 0.1 N NaOH solution. Copper sulfate heptahydrate, cobalt II chloride, and nickel II chloride hexahydrate salts were used to prepare the initial concentrations of each heavy metal ion. Experiments were carried out in glass conical flasks that were rapidly shaken at 180 rpm with a laboratory shaker. The adsorption efficiency is obtained using eq 4^7^
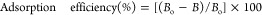
4where *B*_o_ and *B* are the starting and final concentrations, respectively, of each heavy metal ion in M (molar).

## 3 Results and Discussion

### 3.1 Surface Characterization

#### 3.1.1 Scanning Electron Microscopy

**Figure 2:**
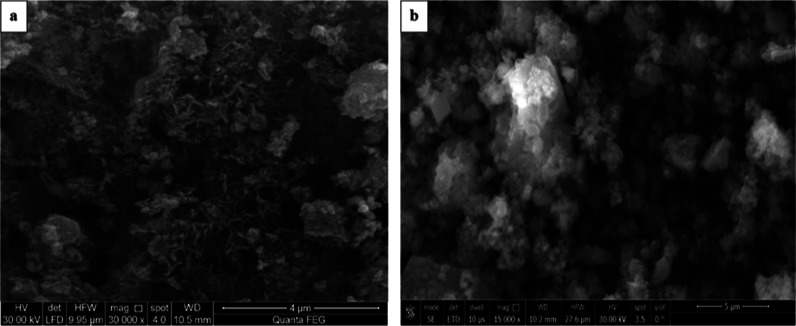
SEM images of the CC (a) and CC-ZnO-Fe_3_O_4_ nanocomposites (b).

The honeycomb structure of (CC) is clearly observed in Figure 2a, confirming the successful carbonization of chitosan. Figure 2b shows that the synthesized nanocomposite (CCZF), which contained spherical particles, was ZnO particles, as was the case for some rectangular flakes of Fe_3_O_4_ that precipitated on the surface of (CC). Furthermore, some agglomeration was observed due to the magnetic properties of Fe_3_O_4_ as well as the polarity and electrostatic attraction of the ZnO particles.

#### 3.1.2 TEM

**Figure 3:**
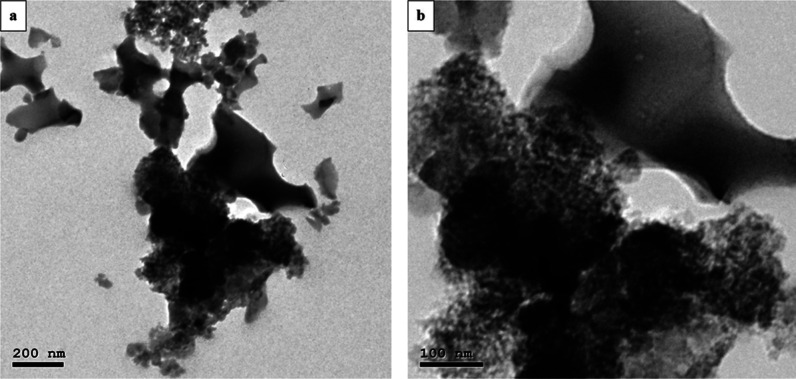
TEM images of the CC-ZnO-Fe_3_O_4_ nanocomposite at 200 nm (a) and 100 nm (b).

Due to the deposition and distribution of Fe_3_O_4_ and ZnO particles on the (CC) surface, agglomerations appeared as nonspherical shapes, as illustrated in Figure 3. Additionally, the darkest areas displayed (CC) (precursor), while the lighter areas revealed that the Fe_3_O_4_ and ZnO particles precipitated on the surface of the (CC).

#### 3.1.3 BET

**Figure 4:**
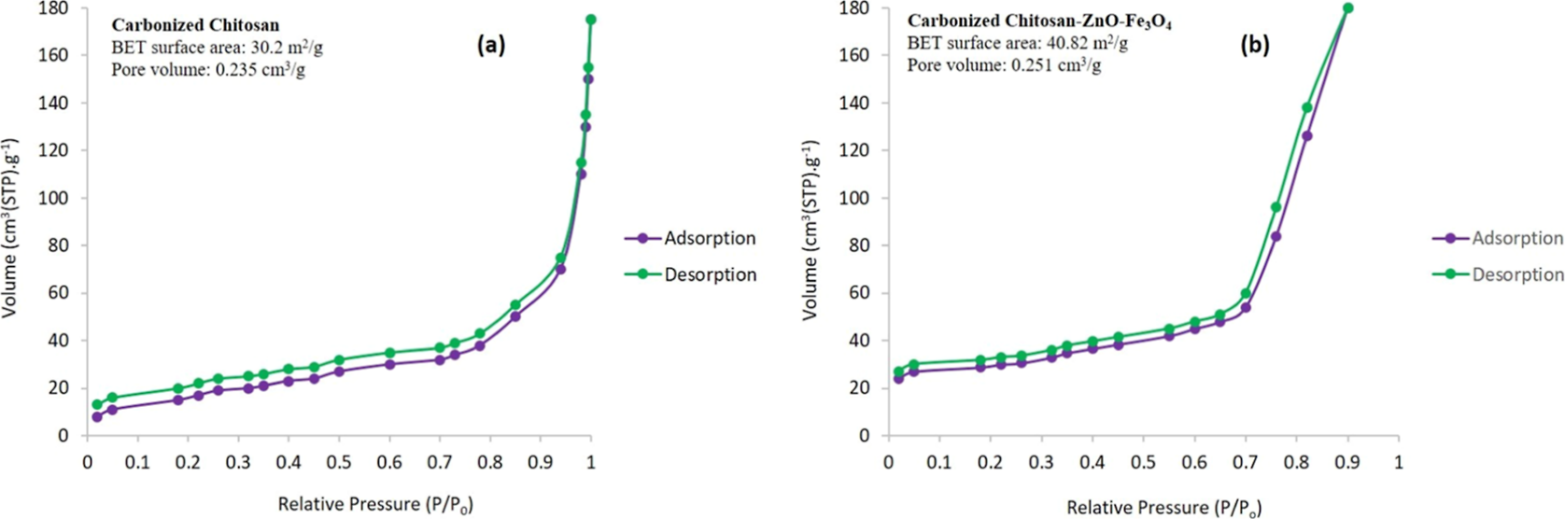
N_2_ adsorption–desorption isotherms of CC (a) and the CC-ZnO-Fe_3_O_4_ nanocomposite (b).

As shown in Figure 4, N_2_ adsorption–desorption analyses of (CC) and the prepared (CCZF) composite were used to determine their specific surface areas and pore volumes. There were 30.2 and 40.82 m^2^/g BET surface areas, as well as cumulative pore volumes of 0.235 and 0.251 cm^3^/g for the (CC) and (CCZF) composites, respectively. Furthermore, the BET analysis indicated that the Fe_3_O_4_ and ZnO particles contributed significantly to increasing the active surface area of the (CC).

#### 3.1.4 XRD

**Figure 5:**
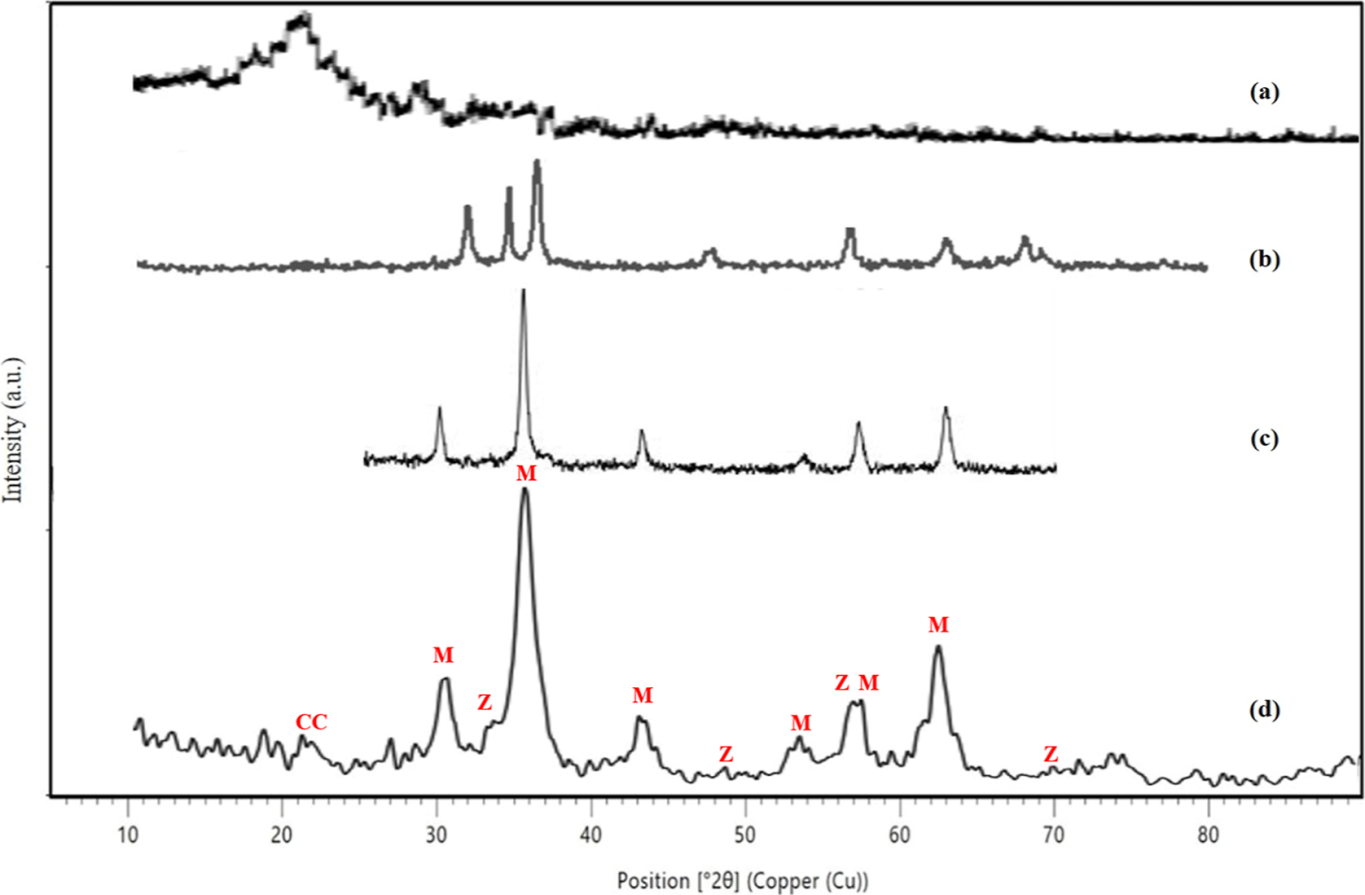
XRD patterns of CC (a), ZnO (b), Fe_3_O_4_ (c), and the CC-ZnO-Fe_3_O_4_ nanocomposite (d): (CC, M: magnetite, and ZnO).

A broad peak could be observed at 2θ ranging from 20° to 23° in Figure 5a, which illustrates the amorphous nature of CC.^19^Figure 5b shows that the ZnO NP peaks appeared at 2θ = 32.5, 33.92, 37, 47.2, 56.9, 67, and 69.5°.^20^ Free Fe_3_O_4_ NPs are observed in Figure 5c, with sharp peaks at 2θ = 30.9, 35.5, 44.1, 53.82, 57.8, and 62.43°.^21^Figure 5d shows the pattern of the prepared composite (CCZF), where the intensity of the (CC) peak decreased substantially and the ZnO peak at 2θ = 37° disappeared due to the high crystallinity of Fe_3_O_4_. All of the Fe_3_O_4_ peaks and the remaining ZnO peaks appeared, confirming the successful precipitation of ZnO and Fe_3_O_4_ on the surface of the CC.

#### 3.1.5 FTIR

**Figure 6:**
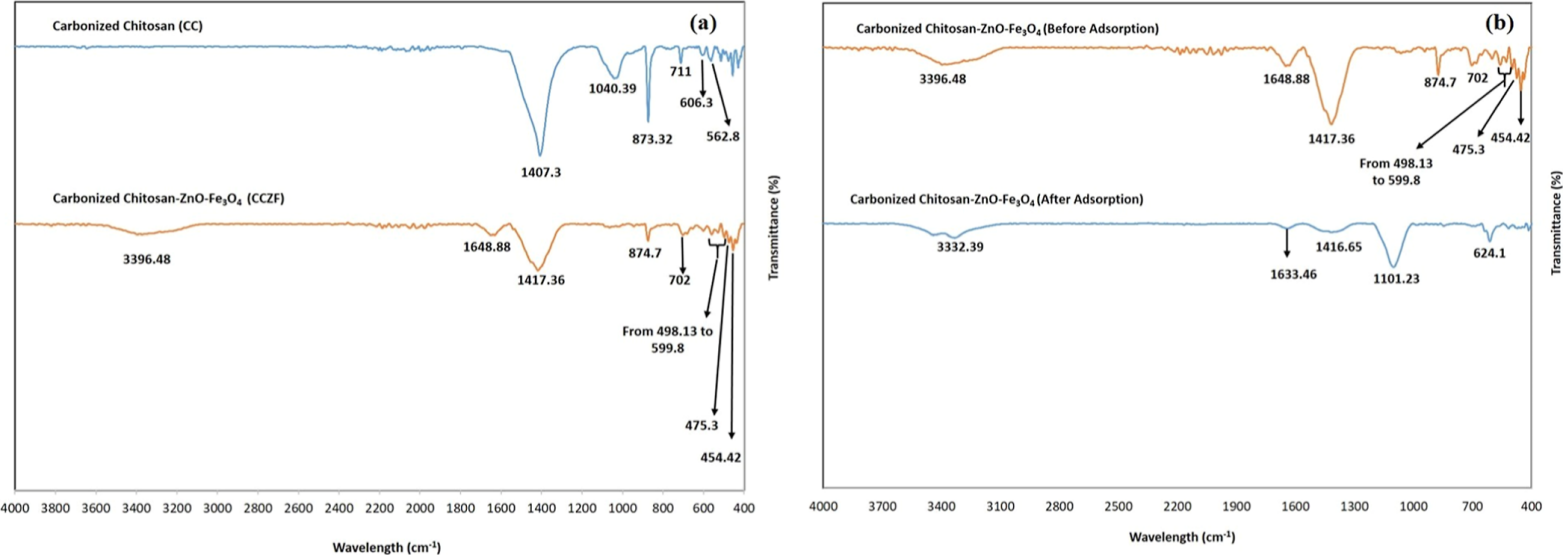
FTIR analysis of CC and CC-ZnO-Fe_3_O_4_ nanocomposites (a) and before versus after adsorption (b).

Figure 6a shows the FTIR band of the (CC) versus the CCZF-synthesized nanocomposite. FTIR analysis was performed at wavelengths ranging between 400 and 4000 cm^–1^. The strong IR bands at 1407.3 and 1040.39 cm^–1^ attributed to the (C–H) bond were attributed to CC.^22^ Additionally, the sharp peaks at 873.32, 711, 606.3, and 562 cm^–1^ corresponded to C=O.^22^ New peaks were observed in the (CCZF) nanocomposite at 1648.88 and 3396.48 cm^–1^, corresponding to the (–OH) group.^23^ These peaks appeared as a result of washing the nanocomposite with distilled water during preparation. Additionally, two new peaks appeared at 475.3 and 454.42 cm^–1^, which were attributed to the (Zn–O) stretching bond.^24^ The disappearance of the IR bands at 1040.39, 606, and 562 cm^–1^ was due to the deposition of ZnO and Fe_3_O_4_ particles on the surface of the precursor (CC). Furthermore, the peaks at 498.13 to 599.8 cm^–1^ were attributed to Fe–O bonds in the crystalline lattice of Fe_3_O_4_.^24,25^ In Figure 6b, the IR spectra of the (CCZF) nanocomposite before and after the sorption of Co^2+^, Ni^2+^, and Cu^2+^ ions from aqueous solution were shown. A new peak at 1633.46 cm^–1^ was observed after the adsorption process, which corresponded to the Ni^2+^ ion.^26^ As a result of adsorption, two new peaks were observed at 624.1 and 1101.23 cm^–1^, corresponding to Cu_2_O and Co^2+^ toxic elements bound to a C–O group, respectively.^27^ Furthermore, a deficiency in peak intensity was clearly observed at 3396.48 and 1417.36 cm^–1^, indicating the successful adsorption of the heavy metal ion mixture from aqueous solution using the newly prepared composite.

#### 3.1.6 Zeta Potential

**Figure 7:**
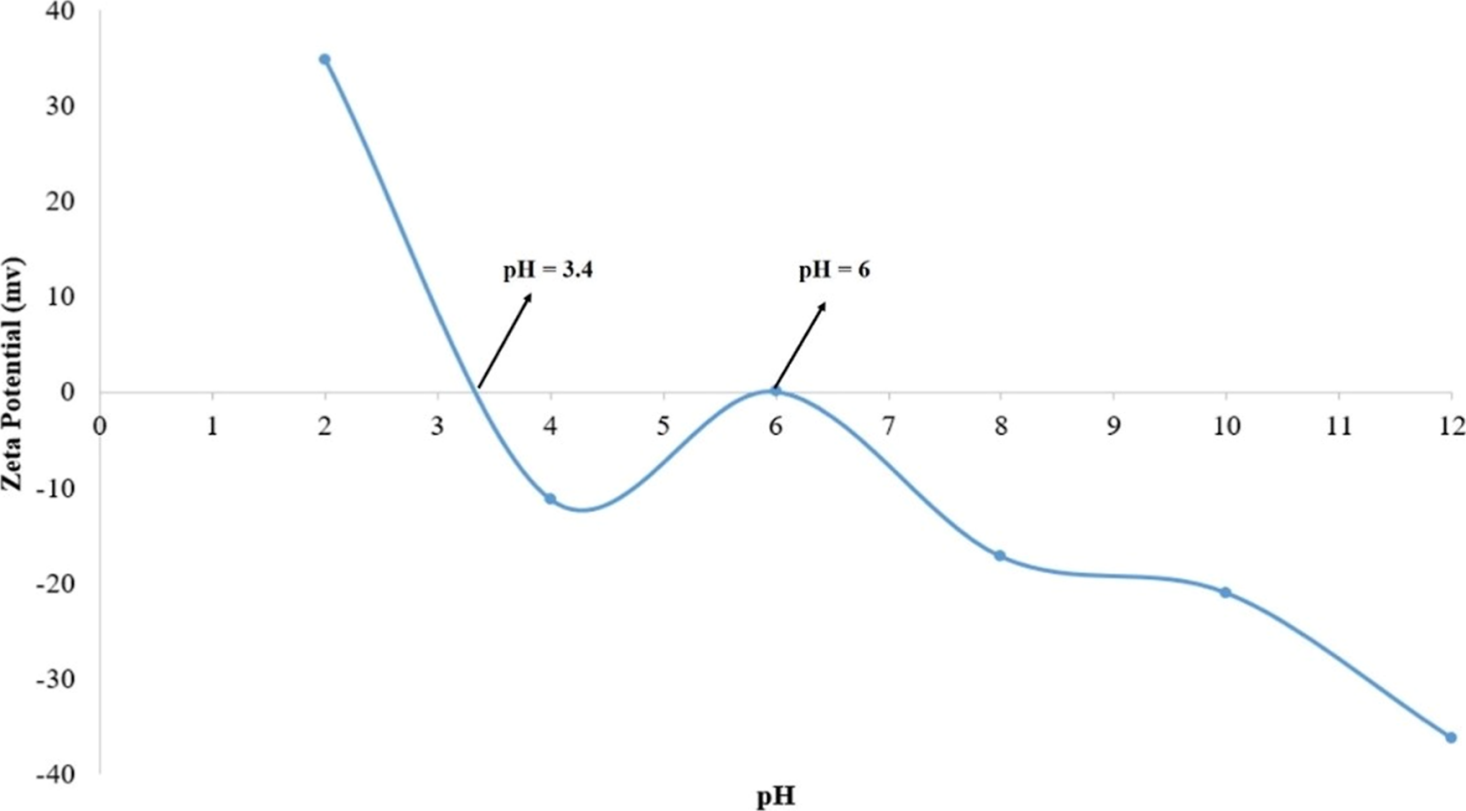
Points of zero charge for the CC-ZnO-Fe_3_O_4_ nanocomposite.

The ZP is a measurement of the difference in potential between the bulk fluid holding particles and the layer of fluid surrounding NP surfaces containing oppositely charged ions.^28^Figure 7 demonstrates that the synthesized nanocomposite had dual pzc at pH = 3.6 and pH = 6. The presence of dual pzc might be due to the complex chemical mechanism of (CC) in the presence of the other composite components ZnO and Fe_3_O_4_. The lower (pzc) at pH = 3.4 was due to the presence of (CC), while the higher pH = 6 was due to the presence of ZnO and Fe_3_O_4,_ which had theoretical (pzc) values of 7.1 and 7.9, respectively.^29,30^

### 3.2 Effect of Contact Time for the Mono-Component System

**Figure 8:**
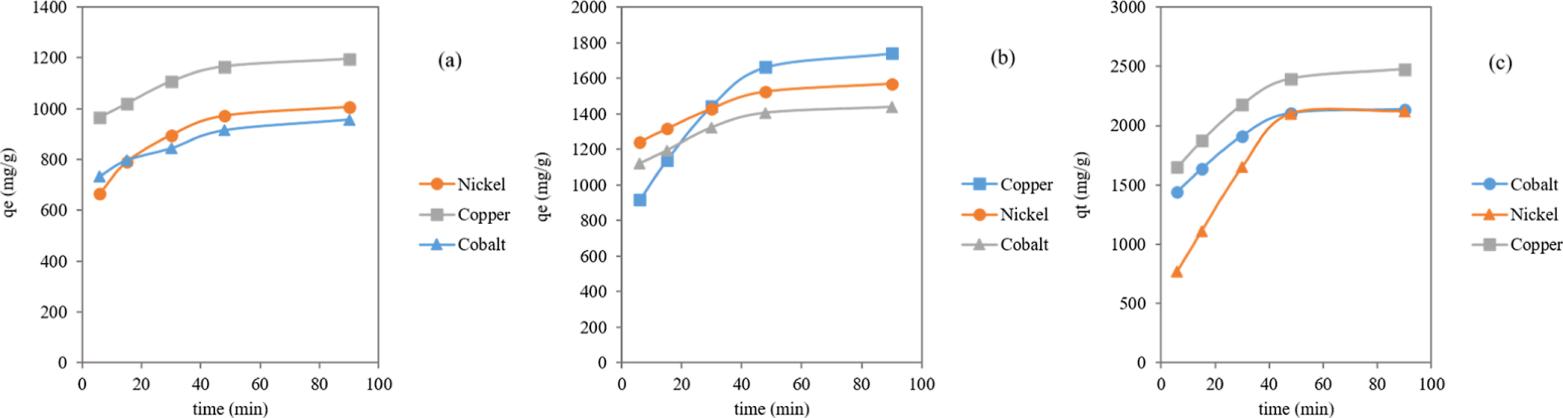
Effect of contact time on the removal of Co^2+^, Ni^2+^, and Cu^2+^ toxic elements at different concentrations 0.05 M (a), 0.075 M (b), and 0.1 M (c).

To ascertain the suitable contact time between the adsorbent and each toxic element in solution, the adsorption capacities were assessed over time.^33^ This study was performed at different levels of time up to 90 min and under experimental conditions of pH 9, initial concentration for each ion of 0.05, 0.075, and 0.1 M, adsorbent dose = 2 g/L, and temperature = 20 °C. The results presented in Figure 8 indicate that the removal of Co^2+^, Ni^2+^, and Cu^2+^ ions likely occurs in two phases. The first phase was relatively swift up to 30 min, while the second phase signified the establishment of equilibrium. This time frame was adequate for achieving equilibrium, thereby enabling the investigation of the parameters that influence the removal of the toxic elements by the analyzed adsorbent. Upon reaching equilibrium, the adsorption rate became constant. The rapid initial phase was presumably due to the high availability of active sites on the surface of the synthesized (CCZF) nanocomposite, while the efficiency of adsorption decreased in the slower second phase as these highly active sites became progressively occupied. For the synthesized (CCZF) nanocomposite, the adsorption of the toxic elements stabilized at a contact time of 90 min. At equilibrium, the adsorption capacities for Co^2+^, Ni^2+^, and Cu^2+^ for 0.05 M each were recorded at 955.3, 1006.4, and 1195 mg/g, respectively. At equilibrium, the adsorption capacities for Co^2+^, Ni^2+^, and Cu^2+^ for 0.075 M each were recorded at 1438.52, 1567.4, and 1739.2 mg/g, respectively. At equilibrium, the adsorption capacities for Co^2+^, Ni^2+^, and Cu^2+^ for 0.1 M each were recorded at 2135.2, 2121.3, and 2473.7 mg/g, respectively. This significant level of adsorption could be attributed to the presence of easily accessible reactive sites on the outer surface of the (CCZF) nanocomposite, which enhanced the removal of the toxic elements during the initial phase.

### 3.3 Study of Adsorption Kinetics of the Ternary-Component System

The process of external diffusion encompasses the transfer of the adsorbate from the bulk solution to the external surface of the adsorbent.^7^ Subsequently, internal diffusion takes place, allowing the adsorbate to traverse the pores within the adsorbent.^7^ The final stage involves the adsorption of the adsorbate onto the active sites of the adsorbent.^7^ The step that exhibits the slowest rate among these three is recognized as the rate-limiting step. To interpret this process, experimental data can be fitted to various kinetic models, which aids in identifying the most plausible adsorption mechanism.^7,31^ The adsorption kinetics of Co^2+^, Ni^2+^, and Cu^2+^ ions by the synthesized (CCZF) nanocomposite were studied using the pseudo-first-order (PFO), pseudo-second-order (PSO), Elovich, intraparticle diffusion, and Boyd plot rules mentioned in previous literature.^7,31−33^ This kinetics study was executed at an operating condition of pH = 9, starting concentration of the metal ion mixture = 0.1, 0.075, and 0.05 M, temperature = 20 °C, and adsorbent amount = 2 g/L. Table 1 represents the parameters of the kinetic models and the correlation coefficients (R^2^), *k*_1_, and *k*_2_ are the rate constants for the PFO and PSO models, respectively.

**Table 1:** Parameters of the PFO and PSO Models

model	concentration (M)	parameters	Co^2+^	Ni^2+^	Cu^2+^
PFO	0.1	R^2^	0.9993	0.9833	0.9996
		*k*_1_ (min^–1^)	3.45 × 10^–5^	1.25 × 10^–5^	5.16 × 10^–5^
	0.075	R^2^	0.9973	0.9929	0.9996
		*k*_1_ (min^–1^)	1.2 × 10^–4^	2.75 × 10^–5^	0.0016
	0.05	R^2^	0.9906	0.9943	0.9978
		*k*_1_ (min^–1^)	1.3 × 10^–4^	1.1 × 10^–4^	1.5 × 10^–4^
PSO	0.1	R^2^	0.9837	0.9709	0.9726
		*k*_2_ (mg/g.min)	0.035	0.0276	0.04
	0.075	R^2^	0.9778	0.9686	0.9754
		*k*_2_ (mg/g.min)	0.029	0.026	0.033
	0.05	R^2^	0.9642	0.955	0.9562
		*k*_2_ (mg/g.min)	0.022	0.015	0.027
Elovich	0.1	R^2^	0.9035	0.9158	0.8394
		β (g/mg)	0.0027	0.0016	0.0024
		α (mg/g/min)	2775.1	1321.7	3063.2
	0.075	R^2^	0.9126	0.9255	0.8652
		β (g/mg)	0.0065	0.007	0.0024
		α (mg/g/min)	3.1Ee-04	1.15 × 10^–5^	519.36
	0.05	R^2^	0.858	0.8502	0.9448
		β (g/mg)	0.008	0.0062	0.0083
		α (mg/g/min)	4879.7	1564	5 × 10^–4^
intraparticle diffusion	0.1	R^2^	0.8949	0.7622	0.7976
		*k*_p_ (min^1/2^/mg)	139.22	242.55	161.02
		c	1115.6	236.54	1264.8
	0.075	R^2^	0.8896	0.6835	0.5976
		*k*_p_ (min^1/2^/mg)	59.3	55.27	161.02
		c	979	1113.3	531
	0.05	R^2^	0.7621	0.5988	0.6976
		*k*_p_ (min^1/2^/mg)	51.77	61.38	47.1
		c	586.6	539.3	844

**Figure 9:**
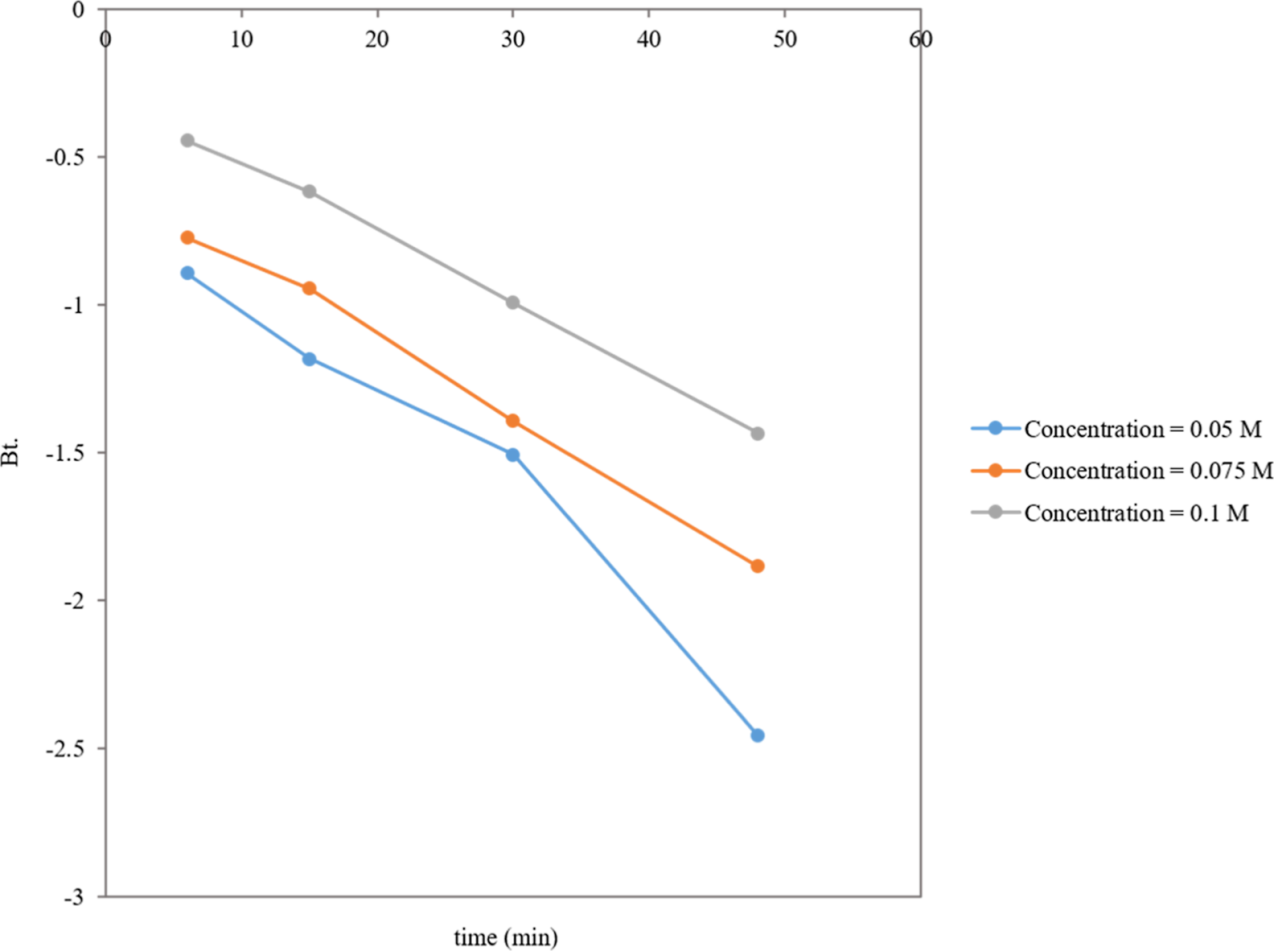
Boyd plot of Co^2+^, Ni^2+^, and Cu^2+^ toxic element adsorption onto the CC-ZnO-Fe_3_O_4_ nanocomposite at different concentrations 0.05, 0.075, and 0.1 M.

Table 1 shows that the current kinetic models do not provide an adequate level of precision for characterizing the removal of toxic elements by using the synthesized (CCZF) nanocomposite. Therefore, the adsorption of the toxic element mixture from aqueous solution using the (CCZF) nanocomposite was well represented by the PFO kinetic model due to its higher *R*^2^ values. It was observed that in the PFO model, the K_1_ adsorption rates of the three toxic elements at different concentrations (0.1, 0.075, and 0.05 M) were higher than those in the PSO model. These observations suggested that the PFO model was the best-fit model due to the higher rate of adsorption of Co^2+^, Ni^2+^, and Cu^2+^ ions from aqueous solutions using the synthesized nanocomposite. Furthermore, this adsorption system relies on physical adsorption. Moreover, the low value of R^2^ in the intraparticle diffusion model suggested that internal diffusion was either sufficiently rapid to not be the rate-limiting step in the adsorption mechanism or that it was not the only factor that limited this adsorption process. Hence, the Boyd plot, as shown in Figure 9, was applied to determine the rate-limiting step where a straight line that did not intersect the origin indicated that the adsorption mechanism was not governed by intraparticle diffusion but the adsorption process influenced by film diffusion or a combination of both film and intraparticle diffusion.^32^

**Figure 10:**
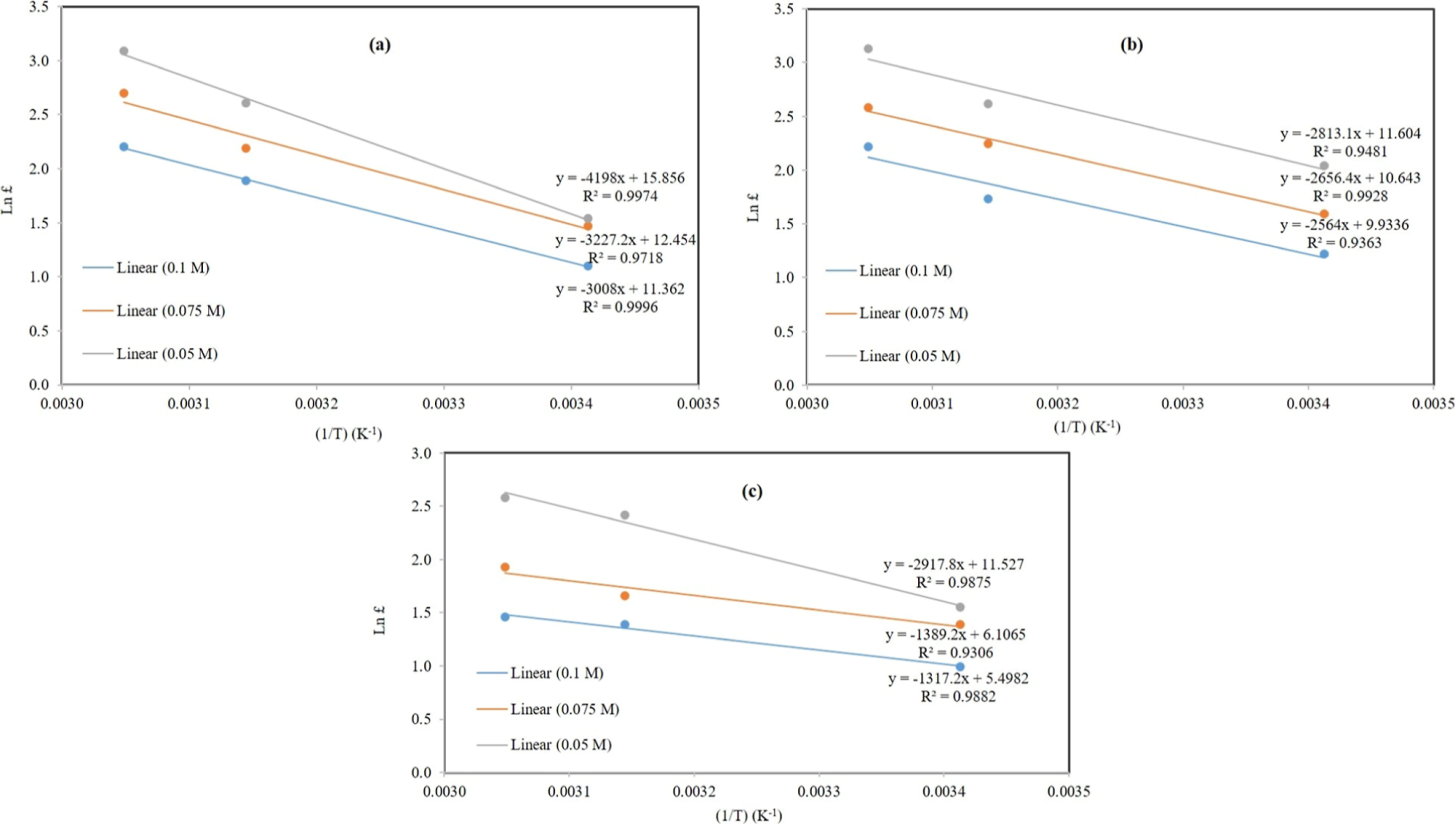
Plots of (1/T) vs ln(k) Ni^2+^ ion (a), Co^2+^ ion (b), and Cu^2+^ ion (c).

### 3.4 Study of the Mono-Component Adsorption Isotherm

The study of adsorption isotherms is vital for gaining insights into the adsorption mechanism, as they provide models that explain the allocation of adsorbed species across solid and liquid phases.^34^ The adsorption of Co ions from aqueous solution using the (CCZF) nanocomposite was investigated using six isotherm models, including Langmuir, Freundlich, Redlich-Peterson (R–P), Temkin, Hill, and Dubinin–Radushkevich (D–R). Their rules were mentioned in the literature.^33−35^ The conditions at which this isotherm study was executed were as follows: adsorbent amount = 3.5 g/L, agitation time of adsorption = 90 min, pH = 9, temperature = 20 °C, and metal ion concentration = 0.05–0.1 M. Table 2 shows that the (R–P) isotherm model was the best-matched model with the experimental data due to its highest *R*^2^ values among the other isotherm models. Therefore, the adsorption mechanism for each single metal ion using the newly prepared nanocomposite was a mix of Langmuir and Freundlich adsorption mechanisms; therefore, it did not follow ideal monolayer adsorption. The maximum adsorption capacities (*q*_max_) of 1428.57, 1767.5, and 1830.7 mg/g for Ni^2+^, Co^2+^, and Cu^2+^, respectively, were achieved using the (CCZF) nanocomposite. Additionally, the activation energies (E) from the (D–R) isotherm model were 0.022, 0.013, and 0.018 kJ/mol for Ni^2+^, Co^2+^, and Cu^2+^, respectively. These values of activation energy (E) were lower than 8 kJ/mol, which indicated that each metal ion adsorption system was controlled by a physical adsorption mechanism.^7^ This conclusion was in alignment with the findings of previous studies.^33^

**Table 2:** Results of the Mono-Component Isotherm Study

model	parameter	Ni^2+^	Co^2+^	Cu^2+^
Langmuir	*R*^2^	0.7916	0.9749	0.9097
	*q*_max_ (mg/g)	1428.57	1767.5	1830.7
	K_L_ (L/mg)	0.0103	0.0049	0.0087
	R_L_ (separation factor)	0.016	0.0335	0.018
Freundlich	*R*^2^	0.6928	0.9441	0.8869
	1/*n*	0.326	0.3745	0.35
	*n*	3.066	2.67	2.861
	K_F_ (L/mg)	159.11	111.92	164.58
R–P	*R*^2^	0.9912	0.9999	0.9921
	A (L/g)	2.54	4.98	11.48
	B (L/mg)	0.0006	0.277	0.034
	β	0.8635	0.177	0.7
Temkin	*R*^2^	0.8828	0.9544	0.8583
	b (J/mol)	6.613	6.063	6.113
	K_m_ (L/g)	0.062	0.037	0.077
Hill	*R*^2^	0.8527	0.9436	0.8162
	qH	1788.5	1867.5	1980.8
	n_H_	0.9335	0.927	0.915
	K_D_	8.23	9.65	7.14
D–R	B (mol^2^/kJ^2^)	1014.3	2793.6	1487.2
	E (kJ/mol)	0.022	0.013	0.018

### 3.5 Adsorption Isotherm Study of the Ternary-Component System

This ternary-component isotherm study was performed based on the rules mentioned in the literature.^36^ The order of strength of the bonds (K_L_) between the ions and the prepared adsorbent from the strongest to the weakest was Cu^2+^ (0.00618 L/mg) > Co^2+^ (0.00184 L/mg) > Ni^2+^ (0.00013 L/mg), as shown in Table 3. Furthermore, the maximum adsorption capacity (*q*_max_) for the simultaneous adsorption of toxic elements from aqueous solution using the synthesized nanocomposite of 3663.4 mg/g was less than the sum of the individual *q*_max_ values for Co^2+^, Ni^2+^, and Cu^2+^ ions (5026.7 mg/g). The reason could be attributed to either the partial overlay of adsorption active sites for Co^2+^, Ni^2+^, and Cu^2+^ ions in the ternary-component system or the occurrence of diverse active sites on the surface of the new composite with different degrees of specificity toward the individual Co^2+^, Ni^2+^, and Cu^2+^ ions. Moreover, it was concluded that the modified Langmuir model was the best-fitted model; thus, the monolayer adsorption mechanism controlled this system according to the (MPSD) values of the Co^2+^, Ni^2+^, and Cu^2+^ ions, as shown in Table 3.

**Table 3:** Results of the Ternary-Component Isotherm Study

model	parameter	*x*_1_	*y*_1_	*z*_1_	*n*	K_F_ (L/mg)	MPSD
extended Freundlich	Ni^2+^	0.6442	–0.9816	0.6392	0.768	28.347	0.5062
	Co^2+^	0.2135	–0.8008	0.1726	2.67	139.15	0.1914
	Cu^2+^	5.2949	–0.8942	5.2794	2.657	140.67	0.2271

modified Langmuir	parameters	η	K_L_ (L/mg)	*q*_max_ (mg/g)	MPSD
	Ni^2+^	4.1 × 10^–5^	0.00013	891.34	0.5181
	Co^2+^	0.00028	0.00184	1269.35	0.8542
	Cu^2+^	0.00069	0.00618	1502.67	0.7240

### 3.6 Effect of Temperature on the Adsorption Process

The adsorption of Ni^2+^, Co^2+^, and Cu^2+^ toxic elements on the nanocomposite (CCZF) was influenced by the temperature. A thermodynamic consideration of the adsorption process is key to determining whether the process is feasible or not. Additionally, whether it is endothermic or exothermic, the values of the thermodynamic parameters (Δ*G*), (Δ*H*), and (Δ*S*) are determined through eqs 5–8^37^
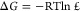
5
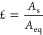
6
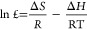
7
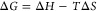
8

Δ*G* is the Gibbs free energy change (kJ/mol K), *R* is the universal gas constant (8.314 J/g mol. K), *T* is the temperature in Kelvin, *A*_s_ is the pollutant concentration on the solid adsorbent at equilibrium (M), *A*_eq_ is the equilibrium pollutant concentration in solution (M), Δ*H* is the enthalpy change (kJ/mol), and Δ*S* is the entropy change (kJ/mol). K and £ are the thermodynamic equilibrium constant. In this research, temperature effects were tested at pH = 9, adsorbent concentration = 2.5 g/L, adsorption time = 90 min, different concentrations (0.05, 0.075, and 0.01 M), and different temperatures (20, 45, and 55 °C), as shown in Table 4.

**Table 4:** Effect of Temperature on the Adsorption of Ni^2+^, Co^2+^, and Cu^2+^ Toxic Elements^a^

temp (K)	conc. (M)	Δ*H* (kJ/mol)			Δ*S* (kJ/mol.K)			Δ*G* (kJ/mol)		
		Ni^2+^	Co^2+^	Cu^2+^	Ni^2+^	Co^2+^	Cu^2+^	Ni^2+^	Co^2+^	Cu^2+^
293	0.1	25.01	21.32	10.95	0.094	0.083	0.046	–2.67	–2.88	–2.44
	0.075							–5.03	–4.95	–3.59
	0.05							–5.98	–5.77	–4.04
318	0.1	26.83	22.09	11.55	0.104	0.088	0.051	–3.51	–3.84	–3.33
	0.075							–6.1	–6.05	–4.59
	0.05							–7.13	–6.94	–5.1
328	0.1	34.9	23.4	24.26	0.132	0.096	0.096	–3.72	–4.88	–3.82
	0.075							–7.02	–7.29	–6.22
	0.05							–8.34	–8.26	–7.18

a The values of Δ*H* and Δ*S* were calculated by plotting (ln £) versus (1/T), as shown in Figure 10, where the slope refers to the value of Δ*H* and the intercept refers to the value of Δ*S*.

Table 4 depicts the endothermic adsorption behavior of Ni^2+^, Co^2+^, and Cu^2+^ toxic elements, respectively, using the (CCZF) nanocomposite due to the positive values of Δ*H*. Moreover, negative values of Δ*G* were observed for this adsorption ternary system, which confirmed the spontaneous nature of this process. Furthermore, the low Δ*H* values ranged from 10.95 to 34.9 kJ/mol <100 kJ/mol for this ternary adsorption system, indicating that the adsorption mechanism was physisorption,^38^ which was in agreement with the results of the ternary-component isotherm study conducted on this adsorption system.

### 3.7 Effect of Interfering Ions on Toxic Element Adsorption

**Figure 11:**
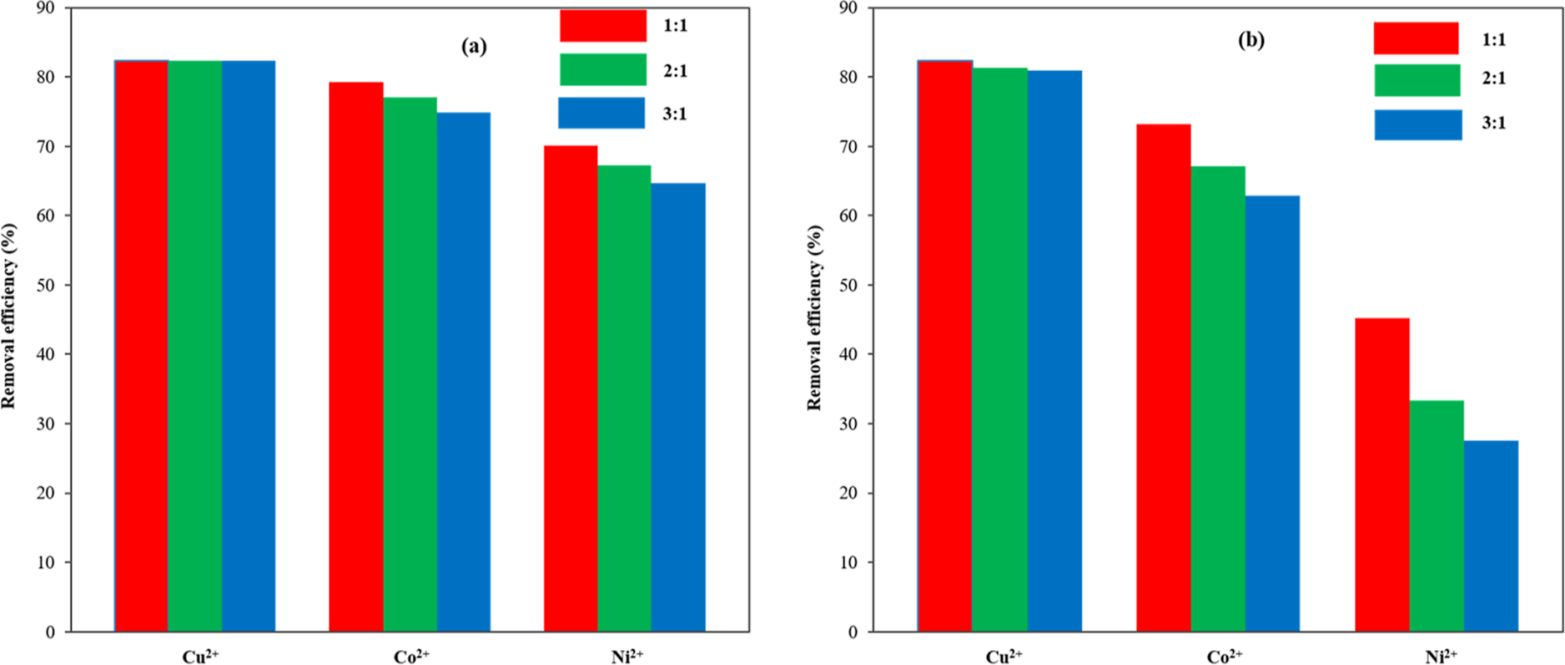
Impact of interfering ions on the adsorption removal efficiencies Na^+^ ions (a) and Ca^2+^ ions (b).

Various metal ions are consistently present in real wastewater. To elucidate the impact of other ions on the removal efficiency of the Cu^2+^, Ni^2+^, and Co^2+^ toxic elements, sodium (Na^+^) and calcium (Ca^2+^) ions were chosen as interfering ions for adsorption experiments. The study also examined the effects of different concentration ratios of these interfering ions on the Cu^2+^, Ni^2+^, and Co^2+^ toxic elements, specifically at ratios of 1:1, 2:1, and 3:1. As illustrated in Figure 11, Na^+^ ions exhibited a minimal impact on the removal efficiencies, even at elevated concentrations. Among the heavy metal ions, the adsorption of Cu^2+^ remained largely unaffected by the presence of Na^+^ and Ca^2+^ ions, suggesting that Cu^2+^ possessed strong resistance to interference. Conversely, the removal efficiency of Ni^2+^ significantly declined from 70.1–45.2% with increasing concentrations of Ca^2+^. This decline could be attributed to the competitive interaction between Ca^2+^ ions and the adsorption sites within the adsorbent material, thereby hindering the adsorption of the Cu^2+^, Ni^2+^, and Co^2+^ toxic elements. The affinity of the synthesized (CCZF) nanocomposite for binding with divalent cations follows the order Cu^2+^ > Co^2+^ > Ni^2+^, indicating that Cu^2+^ was the most adversely affected. Furthermore, the presence of interfering ions with high ionic strength alongside the Cu^2+^, Ni^2+^, and Co^2+^ toxic elements led to a substantial concentration gradient, which diminished the porosity and availability of effective adsorption sites within the (CCZF) nanocomposite, ultimately impacting the adsorption of the Cu^2+^, Ni^2+^, and Co^2+^ toxic elements.

## 4 Design of the Experiments of the Ternary Adsorption System

It was concluded that the best-fit model for prediction of the experimental results was the quadratic model. Equations 9–11 represent the quadratic models for Ni^2+^, Co^2+^, and Cu^2+^ ion adsorption percentages, respectively. The following equations were adjusted by removing insignificant terms to improve their accuracy
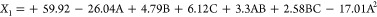
9
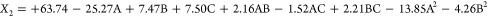
10
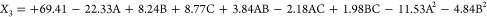
11where X_1_, X_2_, and X_3_ represent the Ni^2+^, Co^2+^, and Cu^2+^ ion adsorption percentages, respectively. A, B, and C are the initial Ni^2+^, Co^2+^, and Cu^2+^ concentrations (M), adsorption agitation time (min), and adsorbent amount (g/L), respectively.

The central composite design (CCD) generated the experimental design matrix for the Ni^2+^, Co^2+^, and Cu^2+^ ion adsorption percentages, adsorption agitation time, and adsorbent amount responses, as shown in Table 5.

**Table 5:** Central Composite Design Experimental Matrix

run	A: concentration of toxic elements (molar)	B: agitation time (min)	C: adsorbent amount (g/L)	Ni^2+^ removal percent (%)	Co^2+^ removal percent (%)	Cu^2+^ removal percent (%)
1	0.1	48	2	19.25	26.65	37.1
2	0.1	90	3.5	25.4	38.11	49.6
3	0.075	48	2	60.05	63.51	69.15
4	0.1	90	0.5	15.23	20.2	33.96
5	0.05	48	2	66.2	73.83	79.43
6	0.075	48	2	60.05	63.51	69.15
7	0.075	6	2	46.42	50.66	56.43
8	0.075	90	2	65.34	69.01	73.48
9	0.05	90	0.5	55.73	66.01	65.47
10	0.1	6	3.5	8.38	13.5	23.32
11	0.075	48	2	60.05	63.51	69.15
12	0.05	6	0.5	62.22	58.88	62.47
13	0.05	6	3.5	70.65	74.03	78.9
14	0.1	6	0.5	4.55	7.97	12.23
15	0.075	48	2	60.05	63.51	69.15
16	0.075	48	0.5	52.67	56.18	60.83
17	0.075	48	2	60.05	63.51	69.15
18	0.05	90	3.5	78.44	86.43	93.19
19	0.075	48	3.5	68.72	72.21	77.67
20	0.075	48	2	60.05	63.51	69.15

### 4.1 Impacts of Process Variables

#### 4.1.1 Nickel Ion Adsorption Model

Response surface curves were used to provide a complete characterization of the system, with several inputs and outputs.

##### 4.1.1.1 Concentration Impact of Co^2+^, Ni^2+^, and Cu^2+^ Toxic Elements

**Figure 12:**
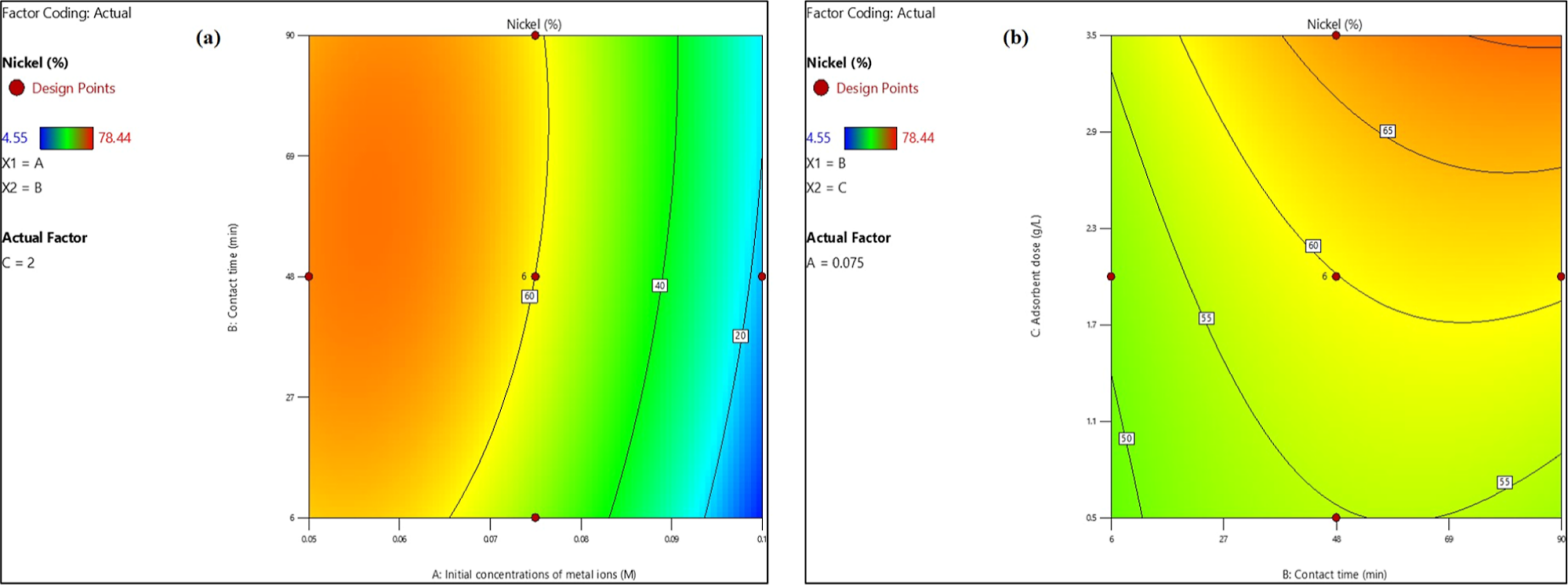
Impact of heavy metal ion concentration (a) and the adsorbent amount (b) on Ni^2+^ ion removal percentage.

The regression analysis presented in eq 9 revealed that the coefficient associated with the initial concentration of toxic elements Co^2+^, Ni^2+^, and Cu^2+^ demonstrated an inverse relationship with the percentage of Ni^2+^ ions adsorbed. This phenomenon can be attributed to a reduction in the availability of active sites on the adsorbent as the concentrations of Co^2+^, Ni^2+^, and Cu^2+^ increased. As illustrated in Figure 12a, increasing the initial concentrations of Co^2+^, Ni^2+^, and Cu^2+^ ions from 0.065 to 0.095 M led to a decline in the nickel ion removal efficiency, dropping from 60 to 20%. This pattern was consistent across various agitation times, maintaining a pH of 9 and an adsorbent dosage of 2 g/L”.

##### 4.1.1.2 Impact of Adsorbent Amount

The regression analysis presented in eq 9 indicated a direct proportional relationship between the amount of adsorbent used and the percentage of nickel ion adsorbed. As the quantity of adsorbent increased, the specific surface area also expanded, leading to a greater number of active sites available for the adsorption of Ni^2+^ ions. Figure 12b illustrates that raising the adsorbent dose from 0.5 to 3.5 g/L positively influenced the removal rates of Co^2+^, Ni^2+^, and Cu^2+^ ions, which rose from 50–65% across different agitation times, with a pH of 9 and an initial concentration of the toxic element mixture set at 0.075 M.

#### 4.1.2 Cobalt Ion Adsorption Model

##### 4.1.2.1 Concentration Impact of Co^2+^, Ni^2+^, and Cu^2+^ Toxic Elements

**Figure 13:**
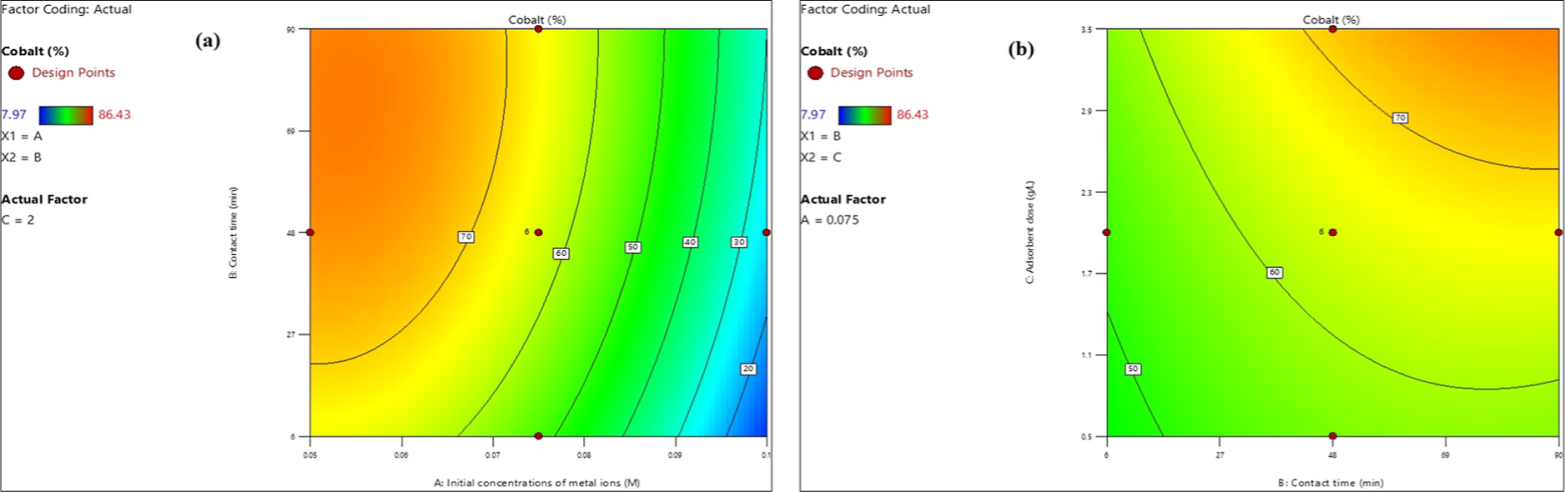
Impact of heavy metal ion concentration (a) and the adsorbent amount (b) on Co^2+^ ion removal percentage.

The regression analysis presented in eq 10 revealed that the initial concentrations of toxic elements (Co^2+^, Ni^2+^, and Cu^2+^) were negative, suggesting an inverse relationship between these concentrations and the percentage of Co^2+^ ion adsorption. This phenomenon can be attributed to a reduction in the number of active sites available on the adsorbent as the concentration of the toxic elements increased. As illustrated in Figure 13a, the percentage of Co^2+^ ion adsorption declined from 70–20% as the initial concentration of the toxic element mixture rose from 0.05 to 0.097 M, under varying agitation durations, with a pH of 9 and an adsorbent dose of 2 g/L.

##### 4.1.2.2 Impact of Adsorbent Amount

The regression analysis presented in eq 10 demonstrated a direct correlation between the dosage of the adsorbent and the percentage of Co^2+^ ions that were adsorbed. Specifically, as the adsorbent dosage increased, there was a corresponding increase in the specific surface area of the adsorbent, which enhanced the availability of active sites for the adsorption of Co^2+^ ions. Furthermore, Figure 13b illustrates that elevating the adsorbent dosage from 0.5 to 3.5 g/L positively influenced the removal efficiency of Co^2+^, Ni^2+^, and Cu^2+^ ions, with the removal percentage increasing from 50–70% across various agitation times at a pH of 9, with an initial concentration of the toxic element mixture set at 0.075 M.

#### 4.1.3 Copper Ion Adsorption Model

##### 4.1.3.1 Concentration Impact of Co^2+^, Ni^2+^, and Cu^2+^ Toxic Elements

**Figure 14:**
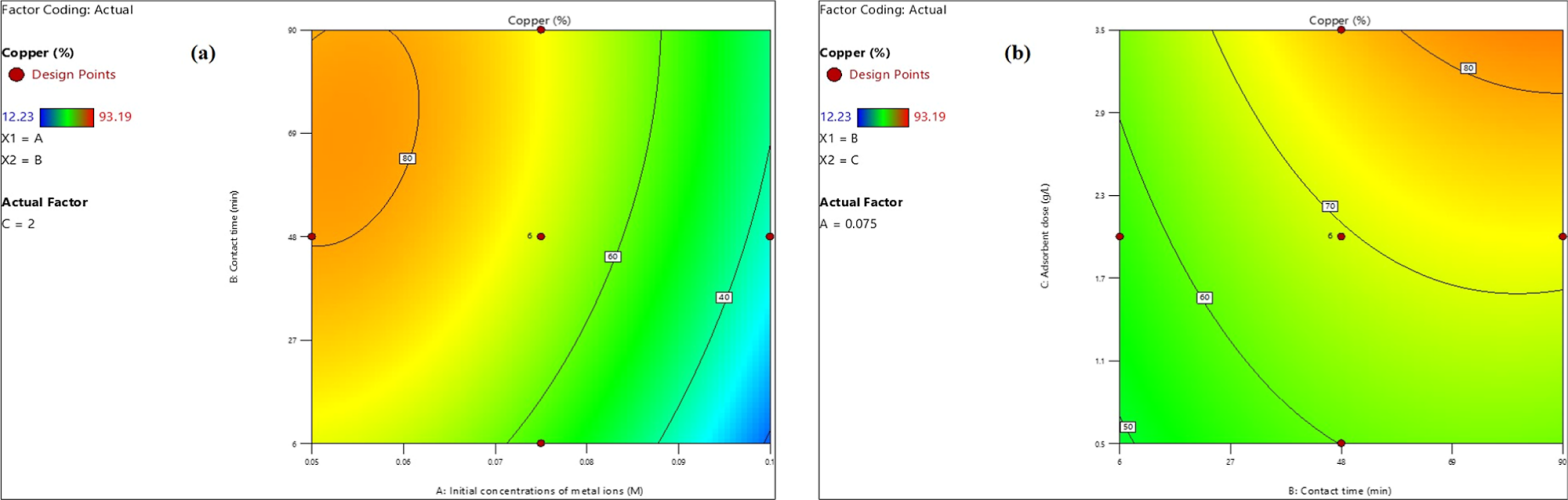
Impact of heavy metal ion concentration (a) and the adsorbent amount (b) on Cu^2+^ ion removal percentage.

The initial concentration of toxic elements Co^2+^, Ni^2+^, and Cu^2+^, as indicated in the regression of eq 11, demonstrated an inverse relationship with the percentage of Cu^2+^ ion adsorption. This phenomenon can be attributed to the reduction in the availability of active sites on the adsorbent as the concentration of heavy metal ions escalated. As illustrated in Figure 14a, the percentage of Cu^2+^ ion adsorption declined from 80–40% as the initial concentration of the toxic element mixture increased from 0.05 to 0.087 M, under varying agitation durations, a pH of 9, and an adsorbent dosage of 2 g/L.

##### 4.1.3.2 Impact of Adsorbent Amount

The analysis conducted through the regression of eq 11 indicated a direct correlation between the dosage of the adsorbent and the percentage of Cu^2+^ ions that were adsorbed. Specifically, as the adsorbent dosage increased, there was a corresponding enhancement in the specific surface area of the adsorbent, which in turn augmented the availability of active sites for the adsorption of Cu^2+^ ions. As depicted in Figure 14b, elevating the adsorbent dosage from 0.5 to 3.5 g/L positively influenced the removal efficiency of the toxic element mixture, increasing from 50–80% across various agitation times, with a pH of 9 and an initial concentration of the toxic element mixture set at 0.075 M.

## 5 Experimental Design and Optimization

Design Expert software (version 13) (Stat-Ease Company, USA), based on response surface methodology-CCD (RSM-CCD), was used to determine the optimal experimental conditions that would result in the maximum removal of toxic elements from aqueous solutions. As shown in Table 6, the optimization targets were chosen to obtain the maximum heavy metal ion adsorption percentage.

**Table 6:** Optimization Limitations

factor	target	minimum	maximum	value
the initial concentration of the toxic elements (M)	minimize	0.05	0.1	
agitation time (min)	maximize	6	90	
adsorbent dose (g/L)	target	0.5	3.5	2.5
Ni^2+^ adsorption (%)	maximize	4.55	78.44	
Co^2+^ adsorption (%)	maximize	7.97	86.43	
Cu^2+^ adsorption (%)	maximize	12.23	93.19	

**Figure 15:**
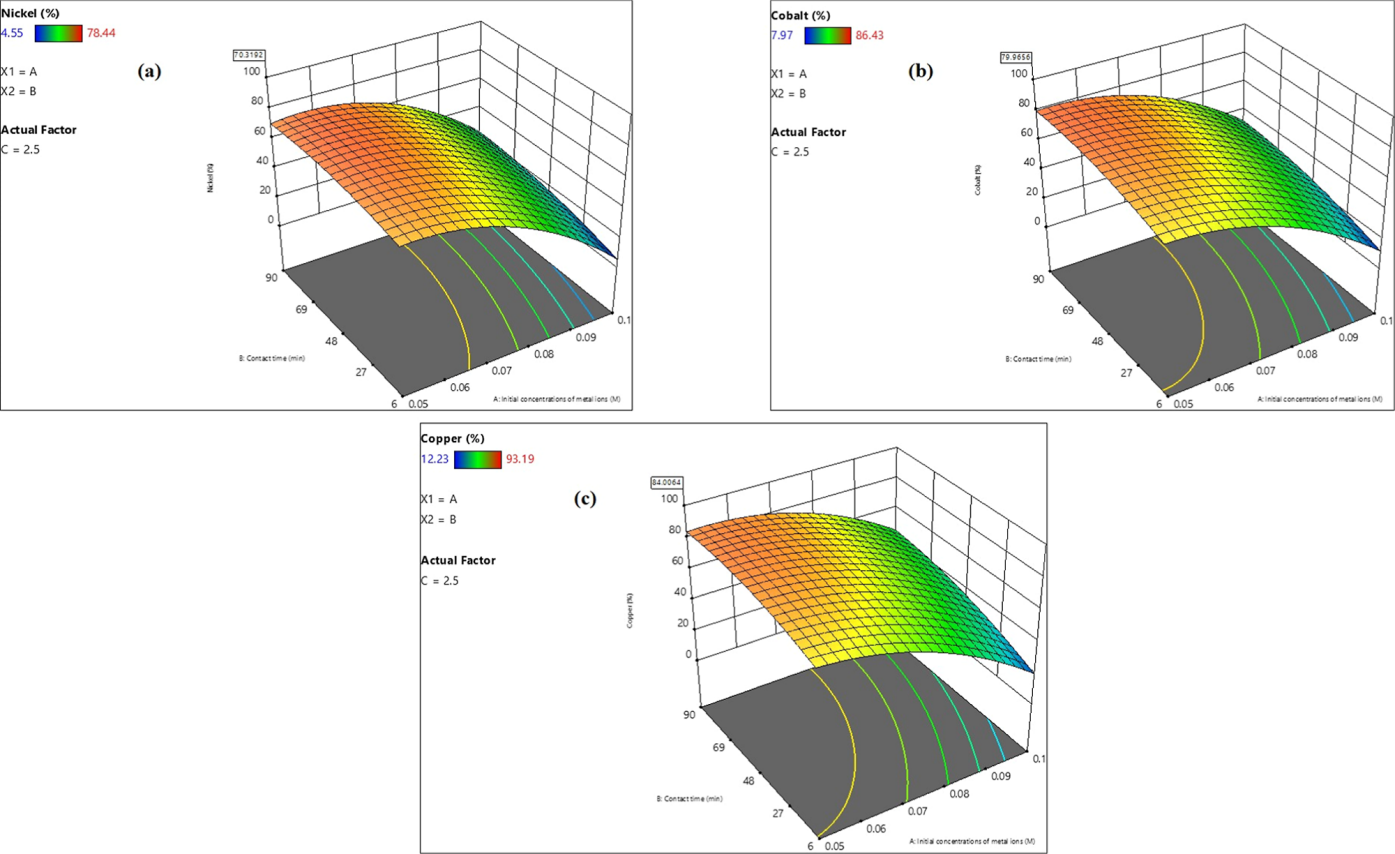
Maximum adsorption percents of Ni^2+^ (a), Co^2+^ (b), and Cu^2+^ (c) ions.

Figure 15 shows that the Ni^2+^, Co^2+^, and Cu^2+^ ion adsorption percentages attained maximum values of 70.13, 79.96, and 84%, respectively, at certain operating conditions of pH = 9, adsorbent amount = 2.5 g/L, starting concentration of the metal ion mixture = 0.05 M, agitation time = 90 min, and temperature = 20 °C.

## 6 Desorption and Recovery

Nitric acid (HNO_3_) or HCl can be used to desorb toxic elements from wastewater or aqueous solutions.^39^ A 0.05 M mixture of toxic elements (Ni^2+^, Co^2+^, and Cu^2+^) at pH = 9 was added to six samples (CCZF), each containing 0.05 g of toxic elements. Following vigorous shaking for 90 min at 20 °C, the composite was filtered, washed in double distilled water, and then dried for 3 h at 40 °C. A desorption solution of 0.5 M HCl was added to each dried composite after adsorption at 20 °C in conical flasks that were shaken at 180 rpm for 15 min, followed by filtration, washing, and drying. The desorption efficiency E_des_ is determined using eq 12(33,39)
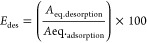
12*A*_eq.adsorption_ is the concentration of toxic elements on the surface of the adsorbent at equilibrium before desorption and *A*_eq.desorption_ is the concentration of toxic elements in the desorption solution at equilibrium.

After washing with deionized water, the spent composite samples were dried in a dryer for 3 h at 40 °C, after which 2.5 g/L was immersed in metal ion solutions at a concentration of 0.05 M, pH = 9, a temperature of 20 °C, and an agitation time of 90 min. Similar experimental conditions were used for the reusability experiments. Equation 13 was used to determine the reusability efficiency for each cycle^39^
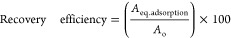
13*A*_eq.adsorption_ is the concentration of toxic elements on the surface of the adsorbent at equilibrium and *A*_o_ is the starting concentration of toxic elements in the aqueous solution.

**Figure 16:**
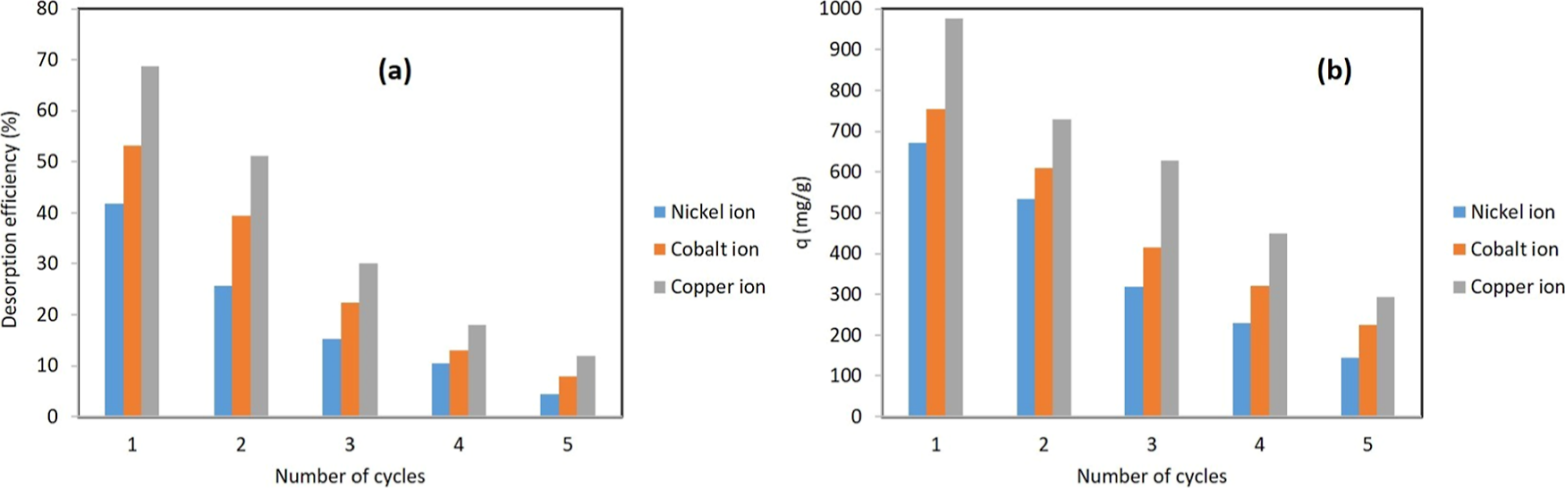
Desorption efficiency (a) and adsorption capacity (b) for Ni^2+^, Co^2+^, and Cu^2+^ heavy metal ions.

Figure 16a shows the desorption efficiencies of the toxic elements, as the highest removal efficiency was observed for the Ni^2+^ ions compared with that for the Co^2+^ and Cu^2+^ ions.

As shown in Figure 16b, the adsorption capacities of the prepared composites for Ni^2+^, Co^2+^, and Cu^2+^ ions decreased with an increasing number of cycles. Furthermore, the order of the adsorption capacity reduction from maximum to minimum was Cu^2+^ > Co^2+^ > Ni^2+^. This was due to the stronger bonding strength of Ni^2+^ to the active sites of the prepared composite compared with that of Co^2+^ and Cu^2+^ ions. Thus, it was difficult for Ni^2+^ ions to be desorbed using HCl (0.5 M). Significant losses in the adsorption capacities of toxic elements were observed after 5 cycles. Consequently, it would be more economically feasible to undertake the adsorption process using a (CCZF) adsorbent, which can recover toxic elements (e.g., Co^2+^, Cu^2+^, and Ni^2+^).

## 7 Comparison with Other Adsorbents

The operating conditions and absorption capacity of this study were compared with those of similar studies. As shown in Table 7, the main novelty of the prepared (CCZF) nanocomposite was that it exhibited greater adsorption capacity of a mixture of Ni^2+^, Co^2+^, and Cu^2+^ toxic elements at higher pollutant concentrations as well as lower adsorbent doses and agitation times than did the other previously documented adsorbents. The great adsorption capacity of the prepared (CCZF) nanocomposite compared with the other adsorbents was due to the presence of chitosan and ZnO, which had great adsorption affinity toward toxic elements of Ni^2+^, Co^2+^, and Cu^2+^ in high capacities. Moreover, the prepared (CCZF) nanocomposite could be separated easily from the solution after the adsorption process due to its magnetic properties.

**Table 7:** Comparison between the (CC-ZnO-Fe_3_O_4_) and the Other Documented Adsorbents

sorbent	pH	agitation time (min)	adsorbent dose (g/L)	initial conc. (mg/L)	(*q*_max_) (mg/g)			*T* (°C)	ref
					Ni^2+^	Co^2+^	Cu^2+^		
vigna radiata husk biomass	8	42	14	10–100	19.88	15.04	11.05	25	(37)
nanostructured layered sodium vanadosilicate (SVS)	7.5	30	0.5	1–200	55.4	70.7	154	25	(40)
chitosan-modified poly(methacrylate)	6	100	0.05	0–200	340	220	195	20	(41)
CCZF	9	90	3.5	2934.5–6355	891.34	1269.3	1502.6	20	this study

## 8 Cost-Effective Analysis

The concept referred to as adsorbent cost performance was represented by a *Ĉ* function.^42^ The *Ĉ* value (in $/mol) quantified the expense associated with producing and utilizing 1 g of an adsorbent to eliminate 1 mol of a chemical species from the aqueous phase, evaluated at the theoretical maximum uptake of that species.^42^ This assessment was conducted by converting all reported adsorption capacities to mol/g and all costs to $/g, which facilitated the calculation of their ratio.^42^ There was significant variability in the costs of adsorbents, with most of them falling within the range of $1 to $200 per mole.^42^ Adsorbents priced below $1 per mole are deemed very economical for their intended use and can be produced on an industrial level, whereas those exceeding $200 per mole are classified as quite costly.^42^Table 8 represents the cost of chemicals used in the production of the (CCZF) nanocomposite.

**Table 8:** Cost of Chemicals Used in the Production of the (CCZF) Nanocomposite

unit	cost ($)
FeCl_3_·6H_2_O (ACS reagent, ≥97%), 100 g	29.35
FeSO_4_·7H_2_O (ACS reagent, ≥99%), 250 g	21.77
(C6H_11_NO_4_)_*n*_ (high molecular weight, degree of quaternization 30–70%), 25 g	55.16
NaOH (ACS reagent, ≥97%, pellets), 500 g	46.3
(CH_3_COO)_2_Zn·2H_2_O (ACS reagent, ≥98%), 500 g	57.42
total cost	210

Table 9 represents the price of chemicals used to produce 4.3 g of the (CCZF) nanocomposite.

- Total chemical price used to synthesize 4.3 g of the (CCZF) nanocomposite = $15.68
- Total chemical price used to synthesize 1 g of the (CCZF) nanocomposite = $3.64/g (CCZF)
- Operating cost to produce 4.3 g of (CCZF), including electricity and water = $4.57
- Operating cost to produce 1 g of (CCZF), including electricity and water = $1.06

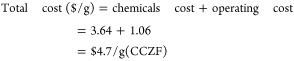
14
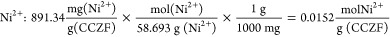
15
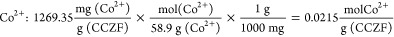
16
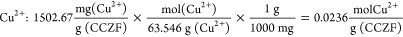
17

**Table 9:** Price of Chemicals Used to Produce 4.3 g of the (CCZF) Nanocomposite

unit	cost ($)
FeCl_3_·6H_2_O (ACS reagent, ≥97%), 5 g	1.46
FeSO_4_·7H_2_O (ACS reagent, ≥99%), 2 g	0.17
(C6H_11_NO_4_)_*n*_ (high molecular weight, degree of quaternization 30–70%), 5 g	11.03
NaOH (ACS reagent, ≥97%, pellets), 10 g	0.92
(CH_3_COO)_2_Zn·2H_2_O (ACS reagent, ≥98%), 18.35 g	2.1
total cost	15.68

Therefore, the cost of 1 g of the synthesized (CCZF) nanocomposite used to remove 1 mol of the Ni^2+^, Co^2+^, and Cu^2+^ toxic elements from aqueous solutions is $78 per mol. This cost is below $200 per mole, suggesting that the synthesized nanocomposite is reasonably priced and can be produced on an industrial scale.

## 9 Future Study

The future study for a novel adsorbent used in the adsorption of heavy metal ions from aqueous solutions can encompass several areas, focusing on its application in real samples, scale-up, and engineering and studying its environmental impact. Here are some potential directions for future research:

(a) Application in Real Samples:

- Test the adsorbent’s effectiveness in real aqueous samples, such as wastewater from industrial processes or contaminated groundwater, to assess practical applicability.
- Conduct studies to understand the competitive adsorption behavior of multiple metal ions present in complex mixtures.

(b) Scale-up and Engineering:

- Explore the scalability of the adsorbent production and its integration into existing wastewater treatment systems.
- Develop prototypes for flow-through reactors or fixed-bed systems to evaluate practical use in large-scale applications.

(c) Environmental Impact Studies:

- Assess the environmental impact of using the adsorbent, including potential leaching and toxicity, to ensure that the solution is safe and sustainable.
- Perform life cycle assessments (LCA) to evaluate the overall sustainability of the adsorbent compared to traditional remediation methods.

## 10 Conclusions

Excess concentrations of toxic elements in industrial wastewater result in extremely harmful effects on human health and marine organisms. Therefore, removal of these toxic elements from wastewater is highly recommended. In this research, a new magnetic nanocomposite (CCZF) was synthesized through a coprecipitation method. This new nanocomposite was used for the simultaneous removal of a mixture of toxic elements (Ni^2+^, Co^2+^, and Cu^2+^) from aqueous solution. To ensure that the synthesis of the new nanocomposite was successful, detailed surface characterization was performed via SEM, TEM, BET analysis, FTIR analysis, and ZP analysis. A kinetic study of the multi-ion adsorption system was implemented, and the PFO model was the best-fitted model for the experimental data. It was concluded that the modified Langmuir ternary system isotherm was the best-matched model according to the experimental data, with maximum adsorption capacities ranging from 891.34 to 1502.67 mg/g for Ni^2+^, Co^2+^, and Cu^2+^ ions. This revealed that the mechanism of this ternary adsorption system involved a single layer. A thermodynamic study was executed on this ternary adsorption system, which reflected the spontaneous and endothermic nature of this adsorption process. Furthermore, the physical adsorption behavior that controls this system was confirmed by the low values of Δ*H* < 100 kJ/mol. The Design Expert software program was used to determine the optimum experimental conditions under which the maximum removal percentages of a mixture of toxic elements (Co^2+^, Ni^2+^, and Cu^2+^) could be achieved. The maximum adsorption percentages of the metal ion mixture from aqueous solution were 70.31, 79.96, and 84%, respectively, at a fixed experimental condition of pH = 9, adsorbent dose = 2.5 g/L, initial concentration of the heavy metal ion mixture (Co^2+^, Ni^2+^, and Cu^2+^) = 0.05 M, agitation time = 90 min, and temperature = 20 °C. The desorption and recyclability of the prepared (CCZF) nanocomposite were evaluated, which illustrated that significant losses in the adsorption capacities of toxic elements were observed after 5 cycles. Therefore, it would be more economically feasible to undertake the adsorption process using the (CCZF) adsorbent, which can recover toxic elements (e.g., Co^2+^, Ni^2+^, and Cu^2+^). In addition, the cost of 1 g of the synthesized (CCZF) nanocomposite used to remove 1 mol of the Ni^2+^, Co^2+^, and Cu^2+^ toxic elements from aqueous solutions is $78 per mol. This cost is below $200 per mole, suggesting that the synthesized nanocomposite is an economical adsorbent with a moderate cost.
